# Therapeutic hypothermia alleviates myocardial ischaemia–reperfusion injury by inhibiting inflammation and fibrosis via the mediation of the SIRT3/NLRP3 signalling pathway

**DOI:** 10.1111/jcmm.17523

**Published:** 2022-08-29

**Authors:** Jing Zhang, Yimei Lu, Peng Yu, Zhangwang Li, Yang Liu, Jun Zhang, Xiaoyi Tang, Shuchun Yu

**Affiliations:** ^1^ Department of Anesthesiology The Second Affiliated Hospital of Nanchang University Nanchang China; ^2^ Department of Metabolism and Endocrinology The Second Affiliated Hospital of Nanchang University Nanchang China; ^3^ The Second Clinical Medical College of Nanchang University Nanchang China

**Keywords:** fibrosis, inflammation, myocardial ischaemia–reperfusion injury, NLRP3, SIRT3, therapeutic hypothermia

## Abstract

Therapeutic hypothermia (TH) may attenuate myocardial ischaemia–reperfusion injury, thereby improving outcomes in acute myocardial infarction. However, the specific mechanism by which TH alleviates MIRI has not been elucidated so far. In this study, 120 healthy male Sprague‐Dawley rats were randomly divided into five groups. Haemodynamic parameters, myocardial infarction area, histological changes and the levels of cardiac enzymes, caspase‐1 and inflammatory cytokines were determined. In addition, the extent of myocardial fibrosis, the degree of cardiomyocyte apoptosis and the expression levels of SIRT3, GSDMD‐N, fibrosis‐related proteins and inflammation‐related proteins were estimated.TH reduced myocardial infarct area and cardiac enzyme levels, improved cardiomyopathic damage and haemodynamic indexes, and attenuated myocardial fibrosis, the protein expression levels of collagen I and III, myocardial apoptosis, the levels of inflammatory cytokines and inflammation‐related proteins. Notably, the immunofluorescence and protein expression levels of SIRT3 were upregulated in the 34H+DMSO group compared to the I/R group, but this protective effect was abolished by the SIRT3 inhibitor 3‐TYP. After administration of Mcc950, the reversal effects of 3‐TYP were significantly abolished, and TH could protect against MIRI in a rat isolated heart model by inhibiting inflammation and fibrosis. The SIRT3/NLRP3 signalling pathway is one of the most important signalling pathways in this regard.

## 1 INTRODUCTION

Myocardial ischaemia at the distal site of a coronary artery occlusion leads to acute myocardial infarction (AMI). AMI is one of the most common causes of morbidity, hospitalization and mortality worldwide.^1^ If ischaemia cannot be relieved in time, the myocardium is replaced by fibrous scar tissue, which affects myocardial contractility. If the scar is large, the overall left ventricular contraction function is impaired, leading to progressive chronic heart failure.^2^ It is urgent to restore blood flow to the ischaemic myocardium and reperfusion. However, during reperfusion, myocardial injury can be aggravated by various mechanisms, including reperfusion injury.^3^

Myocardial ischaemia–reperfusion injury (MIRI) induces an aseptic inflammatory response, which triggers the nucleotide‐binding oligomerization domain (NOD)‐, leucine‐rich repeat (LRR)‐ and pyrin domain‐containing 3 (NLRP3) inflammasome through locally released danger‐associated molecular patterns (DAMP), leading to further damage.^4^ NLRP3 is a sensor that links injury and inflammation. Several common inflammatory diseases can activate the innate immune cytoplasmic signal receptor NLRP3. The NLRP3 nucleus can form inflammasomes, leading to caspase 1 mediated interleukin‐1β (IL‐1β) family cytokines that are proteolytically activated and induce inflammatory and pyroptotic cell death.^5^ Sandager et al.^6^ found in an isolated Langendorff I/R mouse model that the area of myocardial infarction was smaller in mice with NLRP3 gene silencing. Recombinant Sirtuin 3 (SIRT3) is a mitochondrial deacetylase. Recent reports have shown that SIRT3 is involved in the protection against a variety of heart diseases. SIRT3 can modify mitochondrial antioxidant superoxide dismutase 2 (SOD2) to reduce oxidative stress and prevent the development of cardiac hypertrophy.^7^

SIRT3 can activate autophagy, and deacetylated forkhead box O 3α (FOXO3α) can activate the expression of a variety of autophagy genes, thereby protecting cells from apoptosis. SIRT3 induces the initiation and activation of the PINK1‐Parkin autophagy pathway to inhibit cell death.^8^ SIRT3‐deficient mice develop cardiac hypertrophy and interstitial fibrosis after various hypertrophy stimulations because SIRT3 deacetylates and activates FOXO3α, which increases the transcription of antioxidant genes, thereby inhibiting the production of reactive oxygen species (ROS) in stimulated cells.^9^ Experiments have shown that melatonin can induce SIRT3/FOXO3α/parkin‐mediated mitochondrial autophagy and reduce the production of mitochondrial ROS, thereby reducing the activation of NLRP3 inflammasomes in macrophages.^10^, ^11^

Therapeutic hypothermia (TH) is widely used as a cardioprotective treatment for cardiac arrest. TH treatment for patients who are still in a coma after resuscitation from cardiac arrest can improve the survival rate and neurological prognosis.^12^, ^13^ Our previous experiments have shown that TH at 34°C has a protective effect on I/R injured hearts in rat isolated heart models, and it is mediated by phosphatidylinositol 3′‐kinase (PI3K) and nitric oxide (NO).^14^

In summary, we assume that TH can alleviate MIRI by inhibiting inflammation and fibrosis via the mediation of the SIRT3/NLRP3 signalling pathway. Therefore, in this study, we used the Langendorff isolated cardiac perfusion model to observe the molecular expression level of the SIRT3/NLRP3 signalling pathway and understand the cardioprotective mechanism of TH.

## 2 MATERIALS AND METHODS

All experiments were approved by the Institutional Animal Care and Use Committee of Nanchang University.

### 2.1 Animals

A total of 120 healthy male Sprague‐Dawley rats (50–60 days old) weighing 250–300 g were included in this study. All rats were kept in individual cages to keep the breeding environment consistent at a room temperature of approximately 22°C to 24°C. Rats were prevented from receiving strong light and strong noise stimulation to maintain the normal circadian rhythm and were given free access to food and water.

### 2.2 Experimental protocol

**FIGURE 1:**
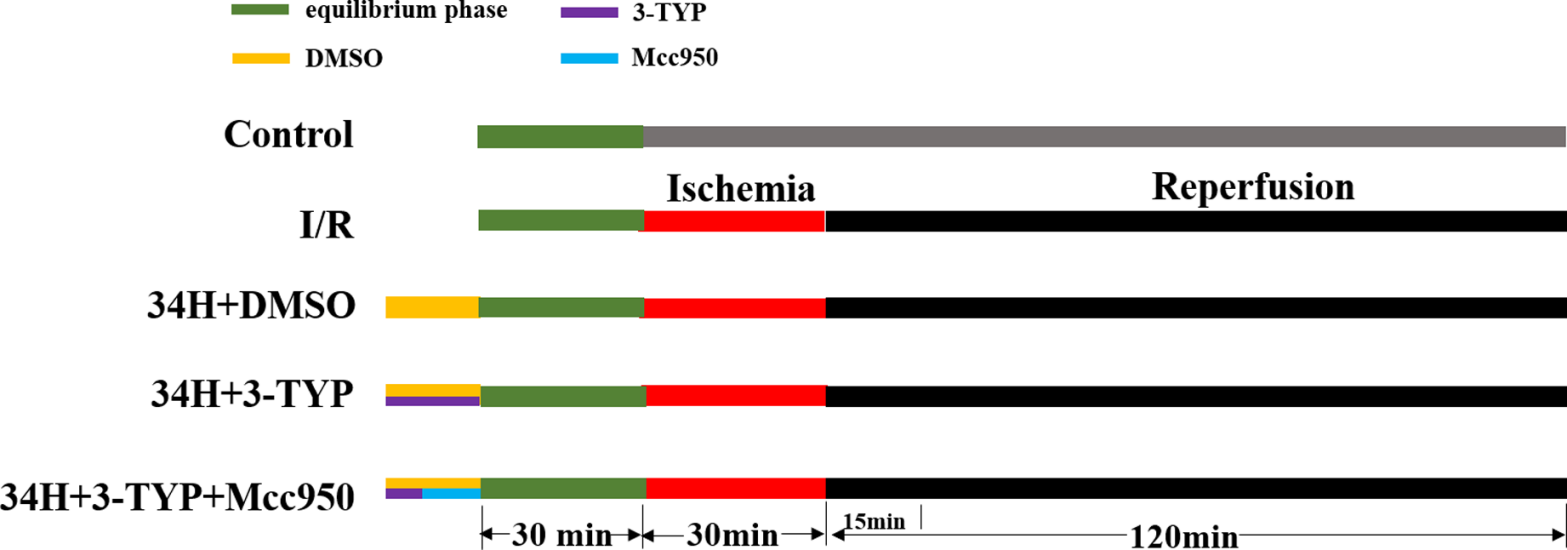
Experimental design. The green area represents the equilibrium phase; the red area represents ischaemia time; the black area represents reperfusion time; the yellow area represents DMSO (50 mg/kg, i.p.); the purple area represents 3‐TYP (50 mg/kg, i.p.); and the blue area represents Mcc950 (20 mg/kg, i.p.).

As shown in Figure 1, after the isolated heart was placed on the Langendorff device, a 30‐min equilibrium period was required to continue the subsequent ischaemia–reperfusion experiment. (1) Control: Rats did not receive any treatment. (2) I/R: The rat hearts were subjected to ischaemia for 30 min, and then reperfusion with 37°C K‐H solution for 2 h. (3) 34H+DMSO: rats were pretreated with DMSO (50 mg/kg, i.p., the solvent of 3‐TYP and Mcc950), and then equilibrium phase for 30 min; the rat hearts were subjected to ischaemia for 30 min and reperfusion with 34°C K‐H solution for 2 h. (4) 34H+3‐TYP: Besides the treatment measures of group 34H+DMSO, rats were pretreated with 3‐TYP (50 mg/kg, i.p.).^15^ (5) 34H+3‐TYP+Mcc950: Besides the treatment measures of group 34H+3‐TYP, rats were pretreated with Mcc950 (20 mg/kg, i.p.).^16^

### 2.3 Langendorff isolated heart perfusion model

Rats were anaesthetised, and then, the rat hearts were quickly excised and mounted on a modified non‐circulating Langendorff apparatus via aorta cannulation for retrograde perfusion at constant pressure (80 mmHg) with Krebs–Henseleit (K‐H) buffer (solution configuration: NaCl 118.0 mmol/L, KCl 4.8 mmol/L, KH_2_PSO_4_ 1.2 mmol/L, NaHCO_3_ 25.0 mmol/L, MgSO_4_ 1.2 mmol/L, CaCl_2_ 2.5 mmol/L, glucose 11.0 mmol/L and pH 7.35–7.45). The K‐H buffer was gassed with 95% O_2_‐5% CO_2_, and the temperature was maintained at 37°C. A small latex balloon connected to a cuff pressure transducer was inserted into the left ventricle through the mitral valve to monitor the rat haemodynamic indexes (Heart rate, HR; left ventricular peak pressure, LVSP; left ventricular end‐diastolic pressure, LVEDP; maximal rate of the increase/decrease of left ventricular pressure, ±dp/dtmax). Haemodynamic indices were recorded at the end of 30 min (T0, the baseline value), and the time points for reperfusion were 30 min (T1), 60 min (T2), 90 min (T3) and 120 min (T4). If the heart developed refractory ventricular fibrillation or frequent arrhythmia during this equilibrium period, or if the software showed LVSP < 75 mmHg or HR < 180 beats/min, the heart was discarded. At the end of reperfusion, the rat hearts were quickly removed for subsequent testing.

### 2.4 Myocardial infarction area measurement

One gram of triphenyl tetrazolium chloride (TTC) was dissolved in 100 ml of PBS phosphate buffer to prepare a 1% TTC solution, which was stored in the dark at 4°C for later use. The hearts were removed after 2 h of reperfusion, washed with 4°C PBS phosphate buffer (pH = 7.4), immediately placed in a −20°C refrigerator, frozen and fixed for 80 min. After taking them out, a self‐made blade was used to slice the heart from the apex to the base into five slices approximately 2 mm thick. The heart tissues were placed in a pre‐configured 1% TTC and incubated in a 37°C incubator in the dark for 20 min. After 24 h, the infarcted area was stained off‐white and the non‐infarcted area was the original pink area of the heart. The area method was used to determine the infarct area. Image‐ProPlus image‐processing software was used to calculate the area of each part. The calculated area of myocardial infarction (%) = grey area/total area of myocardial slice × 100%.

### 2.5 Estimation of myocardial CK‐MB, LDH and cTnl levels

After 2 h of reperfusion, serum samples were isolated from blood samples by centrifugation at 3000 rpm for 10 min at 4°C. The levels of serum cardiac markers CK‐MB, cTnl and the enzyme lactic dehydrogenase (LDH) were measured using commercially available CK‐MB, cTnl and LDH kits (Nanjing Jiancheng Bioengineering Institute, Nanjing, China).

### 2.6 Myocardial HE staining to detect the degree of myocardial damage

After 2 h of reperfusion, heart tissues were immediately removed, fixed in 10% formalin and embedded in paraffin. Haematoxylin–eosin (H&E) staining was performed on 3–5 μm sections of cardiac tissue cut from 10% formaldehyde solution fixed, paraffin‐embedded blocks (*n* = 3 group). The histopathological changes were examined using an optical microscope.

### 2.7 Myocardial Masson staining to detect myocardial fibrosis

Heart tissue samples were fixed with 4% paraformaldehyde, conventionally dehydrated, embedded in paraffin and subjected to Masson staining. Inflammatory infiltration and fibrosis of the heart section were observed under an optical microscope. After staining, the muscle fibres were red and the collagen fibres were blue.

### 2.8 Cardiomyocyte apoptosis analysis

Cardiac tissues at the papillary muscle level were collected for paraffin sectioning. Paraffin sections were stained with the TUNEL assay kit (G3250, Promega, Madison, WI, USA) to examine cardiomyocyte apoptosis, according to the manufacturer's instructions. Images of cardiomyocyte apoptosis were captured using a fluorescence microscope (Zeiss Ltd., Germany). To assess TUNEL activity, all images were randomly examined under identical conditions. Three hearts (*n* = 3/group) were analysed in each experimental group.

### 2.9 Immunofluorescence to detect the expression level of SIRT3

At the end of reperfusion, the myocardial tissue was quickly removed and the myocardial cells were isolated. The slides with cardiomyocytes were immersed in PBS 3 times and fixed with 4% paraformaldehyde for 15 min, and the steps of washing the slides were repeated; normal goat serum was dropped onto the slides to block them for 30 min. After adding a sufficient amount of diluted primary antibody, each slide was placed in a humid box and incubated overnight at 4°C. Next, slides were immersed in PBST 3 min × 3 times, the extra liquid on the slides was absorbed by the absorbent paper, and then, the diluted fluorescent secondary antibody was added dropwise. After adding DAPI dropwise, slides were incubated for 5 min in the dark to stain the nucleus on the specimen. The excess DAPI was washed off with PBST 5 min × 4 times, and the liquid on the slides was absorbed with absorbent paper. The slides were mounted with a mounting solution containing an anti‐fluorescence quencher, and then, the collected images were observed under a fluorescence microscope.

### 2.10 Immunohistochemistry

Cardiac tissue samples were fixed in 4% paraformaldehyde and placed in a 20% sucrose solution at 4°C overnight. Then, tissue samples were embedded in a wax block and cut into slices about 4 mm thick and mounted on polylysine‐coated glass slides. The deparaffinized and hydrated tissue sections were placed in boiled citrate buffer (pH 6.0) and heated for 10 min, after which they were removed, rinsed twice with distilled water and then with PBS for 5 min × 2 times. Next, 50 μl of 3% H_2_O_2_ was added to the tissue area of each slice, incubated for 10 min at room temperature to block the activity of endogenous peroxidase in the tissue and then rinsed with PBS solution. Then, 50 μl of 0.3% TritonX100 was added dropwise to each slice to increase cell permeability. Goat serum blocking solution was added dropwise at room temperature for 30 min, and the excess liquid was discarded. The serum diluent was added to dilute the primary antibody and placed at 4°C overnight. Antibody was discarded, and the slides were washed with PBS for 5 min × 3 times. The biotin‐labelled secondary antibody was diluted in PBS, incubated at room temperature for 2 h and washed with PBS for 5 min × 3 times. The PBS solution was discarded, and 100 μl of freshly prepared DAB solution was added to the sectioned tissue area. Slides were observed under a microscope for 1–3 min, rinsed with tap water, counterstained with haematoxylin and rinsed with PBS solution for blueing. The tissue sections were dried in gradient alcohol dehydration, soaked in xylene and then sealed with neutral gum using the same procedure as before. Images were acquired using an optical microscope.

### 2.11 Enzyme‐linked immunosorbent assay (ELISA)

The levels of cysteinyl aspartate specific proteinase (caspase 1), inflammatory cytokines interleukin‐1β (IL‐1β), interleukin‐6 (IL‐6) and tumour necrosis factor‐α (TNF‐α) were measured using an ELISA kit (Nanjing Jiancheng Bioengineering Institute, Nanjing, China). The samples to be tested and the standard samples were added to the corresponding enzyme plates and cultured at 37°C for 1 h. After washing with PBS, the chromogenic antibody was added to the enzyme plates and incubated for 30 min. Finally, a solution was added to terminate the reaction. The absorbance at 450 nm was measured using a microplate reader.

### 2.12 Western blot analysis

After 2 h of reperfusion, cardiac tissue samples were collected and cellular protein extracts were prepared. Protein concentrations were determined using a bicinchoninic acid protein assay kit (Beyotime Institute of Biotechnology, Haimen, China). Equivalent amounts of protein (30 mg) were separated by 12% SDS‐polyacrylamide gel electrophoresis and then transferred onto a polyvinylidene fluoride (PVDF) membrane. After blocking with 5% non‐fat milk at room temperature for 1 h, primary antibodies against collagen I (1:1000, rabbit, Cell Signalling Technology), collagen III (1:1000, rabbit, Cell Signalling Technology), SIRT3 (1:1000, rabbit, Cell Signalling Technology), NLRP3 (1:1000, rabbit, Cell Signalling Technology), GSDMD‐N (1:1000, rabbit, Abcam) and ASC (1:1000, rabbit, Cell Signalling Technology) were incubated with PVDF membranes at 4°C overnight. The membranes were incubated with horseradish peroxidase‐conjugated mouse anti‐rabbit IgG secondary antibody at room temperature for 1 h. The signals were visualized using an enhanced chemiluminescence Western blot detection system. To control lane loading, the membranes were probed with anti‐GAPDH (1:1000, rabbit; Cell Signalling Technology). Quantitative analysis of the signals was performed by scanning densitometry and analysed using Image Lab Software. The results from each experimental group are expressed as relative integrated intensity compared to that of Control hearts (*n* = 3/group).

### 2.13 Statistical analysis

The results are shown as mean ± standard deviation ($\bar{x}$ ± SD). Statistical analyses were performed using the GraphPad Prism 8.0 software (GraphPad Software, Inc., San Diego, CA, USA). Comparisons between the groups were analysed using a one‐way analysis of variance (anova) followed by Tukey's multiple comparison post‐test (Tukey's test); *p* < 0.05 was considered statistically significant.

## 3 RESULTS

### 3.1 Therapeutic hypothermia led to reduced myocardial infarct size and preserved cardiac function following I/R in rat isolated heart model but the effect was abolished by inhibition of SIRT3

To evaluate the cardioprotective effects of therapeutic hypothermia on ischaemia–reperfusion‐induced heart injury, TCC and HE staining were performed on heart sections cut from isolated rat hearts and treated with different agents.

**FIGURE 2:**
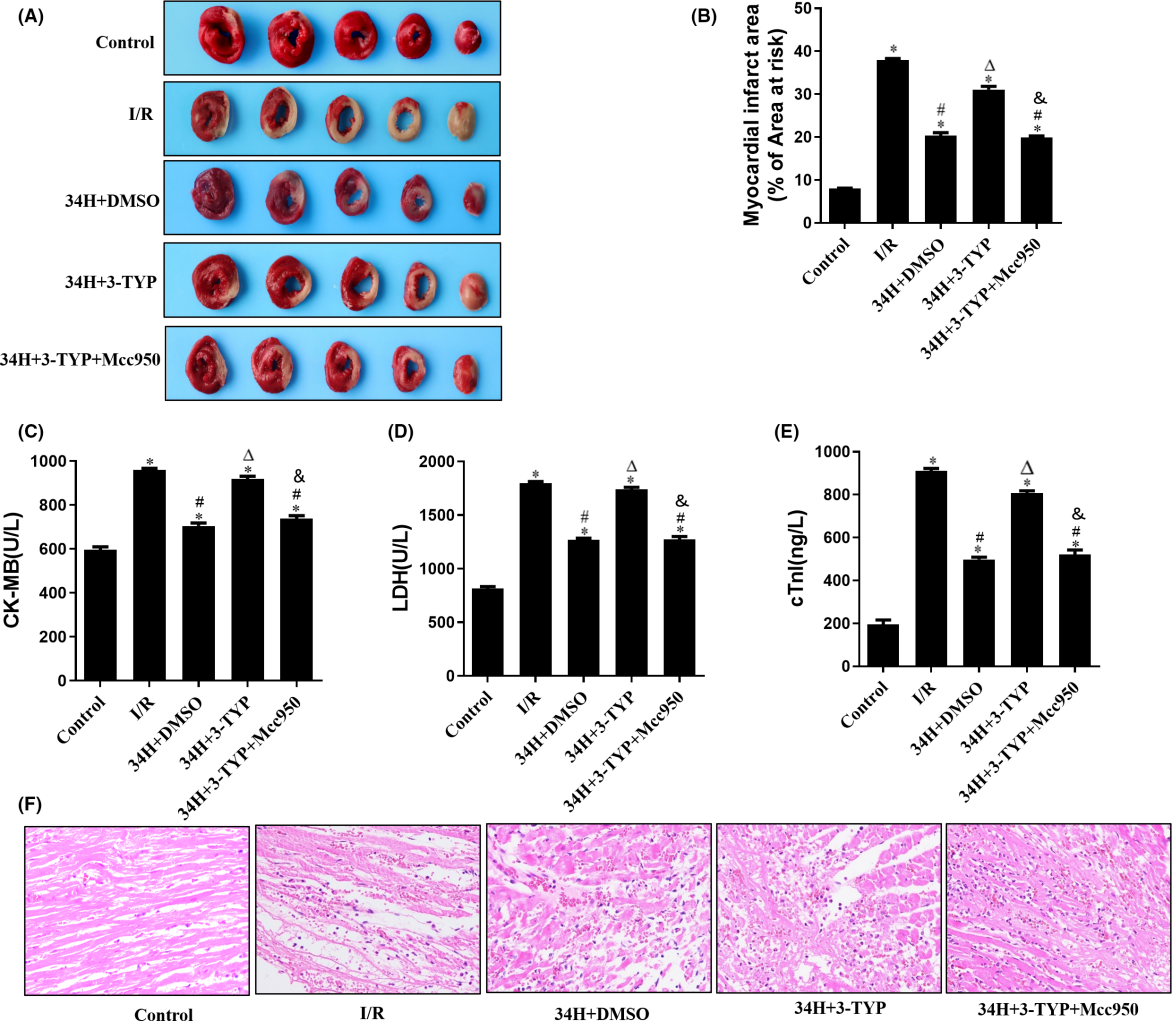
TH ameliorates myocardial infarction and relieves myocardial pathological damage. Therapeutic hypothermia reduces infarct size, lowers serum markers of MI and preserves the myocardium. (A) Representative TTC staining in all groups at the end of reperfusion. (B) Myocardial infarct area % of the area at risk in all groups. (C–E). CK‐MB, LDH and cTnI levels in all groups. (F) Representative haematoxylin and eosin (HE) staining of heart sections from all groups. *p < 0.05 versus control group; #*p* < 0.05 versus I/R group; ^Δ^ *p* < 0.05 versus 34H+DMSO group. ^&^ *p* < 0.05 versus 34H+3‐TYP group.

At the end of reperfusion, TTC staining indicated a rare infarct area in the Control group, while the I/R group showed a significantly increased infarct area compared to the Control group. In addition, TH markedly ameliorated cardiac infarction in the 34H+DMSO and 34H+3‐TYP+Mcc950 groups compare with the I/R group (*p* < 0.05), which could be reversed by 3‐TYP in the 34H+3‐TYP group (*p* < 0.05). Strikingly, compared the 34+3‐TYP with 34H+3‐TYP+Mcc950 group, the reversal effect was abolished by Mcc950 (*p* < 0.05) (Figure 2A,B). In the 34H+DMSO group, the large myocardial infarct size induced by ischaemic reperfusion injury (I/R group) was significantly reversed (*p* < 0.05), and the expression levels of CK‐MB, LDH and cTnI were significantly reduced compared with the I/R group (*p* < 0.05). As before, the reduced myocardial infarct size and decreased expression of CK‐MB, LDH and cTnI (Figure 2C–E) induced by TH in the 34H+DMSO group were significantly reversed in the 34H+3‐TYP group (*p* < 0.05). In addition, compared with 34H+3‐TYP group, the reversal effects of 3‐TYP on myocardial infarct size, CK‐MB, LDH and cTnI were significantly abolished by Mcc950 in the 34H+3‐TYP+Mcc950 group (*p* < 0.05).

HE staining revealed myocardial pathological changes after ischaemia–reperfusion (Figure 2F). In the Control group, the myocardial fibres exhibited a close and regular arrangement, whereas the I/R group showed obvious pathological changes with disorderly arranged and markedly broken myocardial fibres, red‐stained and variously thick transverse bands, dissolved and disappeared myocardial nucleus, and substantial inflammatory cell infiltration. Compared with the I/R group, the myocardial pathological changes in the 34H+DMSO group were significantly milder (*p* < 0.05). Treated with therapeutic hypothermia (34°C), the myocardial fibres were arranged more regularly than the I/R group, and only some of them exhibited broken and slight inflammatory cell infiltration. However, compared with the 34+DMSO group, the improved pathological characteristics of the myocardium could be worsened again by treatment with 3‐TYP in the 34H+3‐TYP group (*p* < 0.05), which could be abrogated by the treatment of Mcc950 compared the 34+3‐TYP with 34H+3‐TYP+Mcc950 group (*p* < 0.05).

Cardiac haemodynamics of isolated hearts in the Langendorff perfusion system were monitored via the MedLab Bio information collection and processing system at five time points after reperfusion. The variation trends of cardiac haemodynamic parameters, including HR, LVSP, LVEDP and ±dp/dtmax in isolated hearts in different groups were almost identical (Table 1). The Control group barely demonstrated changes at a relatively stable level, while a significant decrease in HR, LVSP and +dp/dtmax was observed in I/R control in a time‐dependent manner, which is contrary to LVEDP and −dp/dtmax. Compared with I/R group, these changes in haemodynamic parameters were dramatically reversed by therapeutic hypothermia in the 34H+DMSO group (*p* < 0.05) and compared with the 34H+DMSO group, which could be abolished by 3‐TYP in the 34H+3‐TYP group (*p* < 0.05). However, the abolition of 3‐TYP could also be eased by Mcc950 compared the 34+3‐TYP with 34H+3‐TYP+Mcc950 group (*p* < 0.05).

**TABLE 1:** Myocardial haemodynamics during experiments

Group	Baseline (T0)	Reperfusion			
		30 min (T1)	60 min (T2)	90 min (T3)	120 min (T4)
HR (min^−1^)					
Control	295 ± 16.0	286 ± 8.2	283 ± 13.0	281 ± 11.0	278 ± 7.7
I/R	293 ± 7.8	219 ± 15.0* ^,^ **	204 ± 3.6* ^,^ **	178 ± 9.6* ^,^ **	155 ± 11.0* ^,^ **
34H+DMSO	300 ± 4.6	252 ± 7.2* ^,^ ** ^,^ ***	224 ± 13.0* ^,^ ** ^,^ ***	212 ± 9.5* ^,^ ** ^,^ ***	197 ± 7.6* ^,^ ** ^,^ ***
34H+3‐TYP	293 ± 6.4	206 ± 5.9* ^,^ ** ^,^ ****	211 ± 11.0* ^,^ ** ^,^ ****	186 ± 9.5* ^,^ ** ^,^ ****	168 ± 5.1* ^,^ ** ^,^ ****
34H+3‐TYP+Mcc950	288 ± 10.0	237 ± 6.5* ^,^ ** ^,^ *** ^,^ *****	215 ± 8.1* ^,^ ** ^,^ *** ^,^ *****	192 ± 11.0* ^,^ ** ^,^ *** ^,^ *****	186 ± 7.1* ^,^ ** ^,^ *** ^,^ *****
LVEDP (mmHg)					
Control	7.0 ± 0.40	6.1 ± 0.34	6.0 ± 0.46	6.0 ± 0.42	5.9 ± 0.62
I/R	6.8 ± 0.45	35.0 ± 2.30* ^,^ **	40.0 ± 1.00* ^,^ **	45.0 ± 2.20* ^,^ **	50.0 ± 2.60* ^,^ **
34H+DMSO	7.1 ± 0.44	20.0 ± 0.51* ^,^ ** ^,^ ***	22.0 ± 0.76* ^,^ ** ^,^ ***	25.0 ± 0.36* ^,^ ** ^,^ ***	30.0 ± 0.22* ^,^ ** ^,^ ***
34H+3‐TYP	6.7 ± 0.78	29.0 ± 0.90* ^,^ ** ^,^ ****	34.0 ± 0.67* ^,^ ** ^,^ ****	44.0 ± 1.72* ^,^ ** ^,^ ****	47.0 ± 0.69* ^,^ ** ^,^ ****
34H+3‐TYP+Mcc950	7.1 ± 0.43	21.0 ± 0.72* ^,^ ** ^,^ *** ^,^ *****	23.0 ± 0.77* ^,^ ** ^,^ *** ^,^ *****	27.0 ± 0.24* ^,^ ** ^,^ *** ^,^ *****	31.0 ± 0.79* ^,^ ** ^,^ *** ^,^ *****
LVSP (mmHg)					
Control	99 ± 6.9	95 ± 7.6	91 ± 6.3	89 ± 7.1	87 ± 4.7
I/R	104 ± 6.5	61 ± 5.2* ^,^ **	48 ± 3.8* ^,^ **	42 ± 4.6* ^,^ **	28 ± 5.8* ^,^ **
34H+DMSO	97 ± 8.1	82 ± 3.5* ^,^ ** ^,^ ***	66 ± 4.7* ^,^ ** ^,^ ***	59 ± 4.1* ^,^ ** ^,^ ***	54 ± 3.3* ^,^ ** ^,^ ***
34H+3‐TYP	98 ± 4.7	64 ± 0.9* ^,^ ** ^,^ ****	57 ± 1.5* ^,^ ** ^,^ ****	46 ± 3.3* ^,^ ** ^,^ ****	35 ± 8.6* ^,^ ** ^,^ ****
34H+3‐TYP+Mcc950	99 ± 10.0	82 ± 6.5* ^,^ ** ^,^ *** ^,^ *****	73 ± 8.1* ^,^ ** ^,^ *** ^,^ *****	60 ± 11.0* ^,^ ** ^,^ *** ^,^ *****	52 ± 4.7* ^,^ ** ^,^ *** ^,^ *****
+dp/dtmax (mmHg/s)					
Control	2793 ± 190	2643 ± 194	2608 ± 168	2639 ± 175	2567 ± 182
I/R	2739 ± 175	1774 ± 52* ^,^ **	1452 ± 113* ^,^ **	1165 ± 175* ^,^ **	917 ± 134* ^,^ **
34H+DMSO	2836 ± 182	2402 ± 101* ^,^ ** ^,^ ***	2012 ± 106* ^,^ ** ^,^ ***	1920 ± 95* ^,^ ** ^,^ ***	1818 ± 126* ^,^ ** ^,^ ***
34H+3‐TYP	2776 ± 2124	1819 ± 218* ^,^ ** ^,^ ****	1509 ± 118* ^,^ ** ^,^ ****	1207 ± 80* ^,^ ** ^,^ ****	940 ± 92* ^,^ ** ^,^ ****
34H+3‐TYP+Mcc950	2766 ± 267	2278 ± 121* ^,^ ** ^,^ *** ^,^ *****	1924 ± 79* ^,^ ** ^,^ *** ^,^ *****	1811 ± 77* ^,^ ** ^,^ *** ^,^ *****	1777 ± 73* ^,^ ** ^,^ *** ^,^ *****
–dp/dtmax (mmHg/s)					
Control	−2756 ± 147	−2773 ± 149	−2983 ± 206	−2783 ± 221	−2879 ± 198
I/R	−2636 ± 129	−1755 ± 78* ^,^ **	−2431 ± 163* ^,^ **	−1802 ± 130* ^,^ **	−2240 ± 66* ^,^ **
34H+DMSO	−2630 ± 211	−1488 ± 115* ^,^ ** ^,^ ***	−1991 ± 63* ^,^ ** ^,^ ***	−1471 ± 103* ^,^ ** ^,^ ***	−1959 ± 99* ^,^ ** ^,^ ***
34H+3‐TYP	−2619 ± 123	−1119 ± 62* ^,^ ** ^,^ ****	−1881 ± 94* ^,^ ** ^,^ ****	−1231 ± 115* ^,^ ** ^,^ ****	−1850 ± 87* ^,^ ** ^,^ ****
34H+3‐TYP+Mcc950	−2577 ± 158	−904 ± 66* ^,^ ** ^,^ *** ^,^ *****	−1804 ± 107* ^,^ ** ^,^ *** ^,^ *****	−914 ± 85* ^,^ ** ^,^ *** ^,^ *****	−1743 ± 78* ^,^ ** ^,^ *** ^,^ *****

*Note*: Values are means ± SD (*n* = 6/group).

Abbreviations: HR, heart rate; LVEDP, left ventricular end‐diastolic pressure; LVSP, left ventricular systolic pressure left ventricular systolic pressure; +dp/dtmax, maximal rate of the increase of left ventricular pressure; −dp/dtmax, maximal rate of the decrease of left ventricular pressure.

* *p* < 0.05 versus T0;

** *p* < 0.05 versus control group;

*** *p* < 0.05 versus I/R group;

**** *p* < 0.05 versus the 34H+DMSO group;

***** *p* < 0.05 versus the 34H+3‐TYP group.

### 3.2 Therapeutic hypothermia alleviated myocardial fibrosis caused by I/R in isolated rat models

**FIGURE 3:**
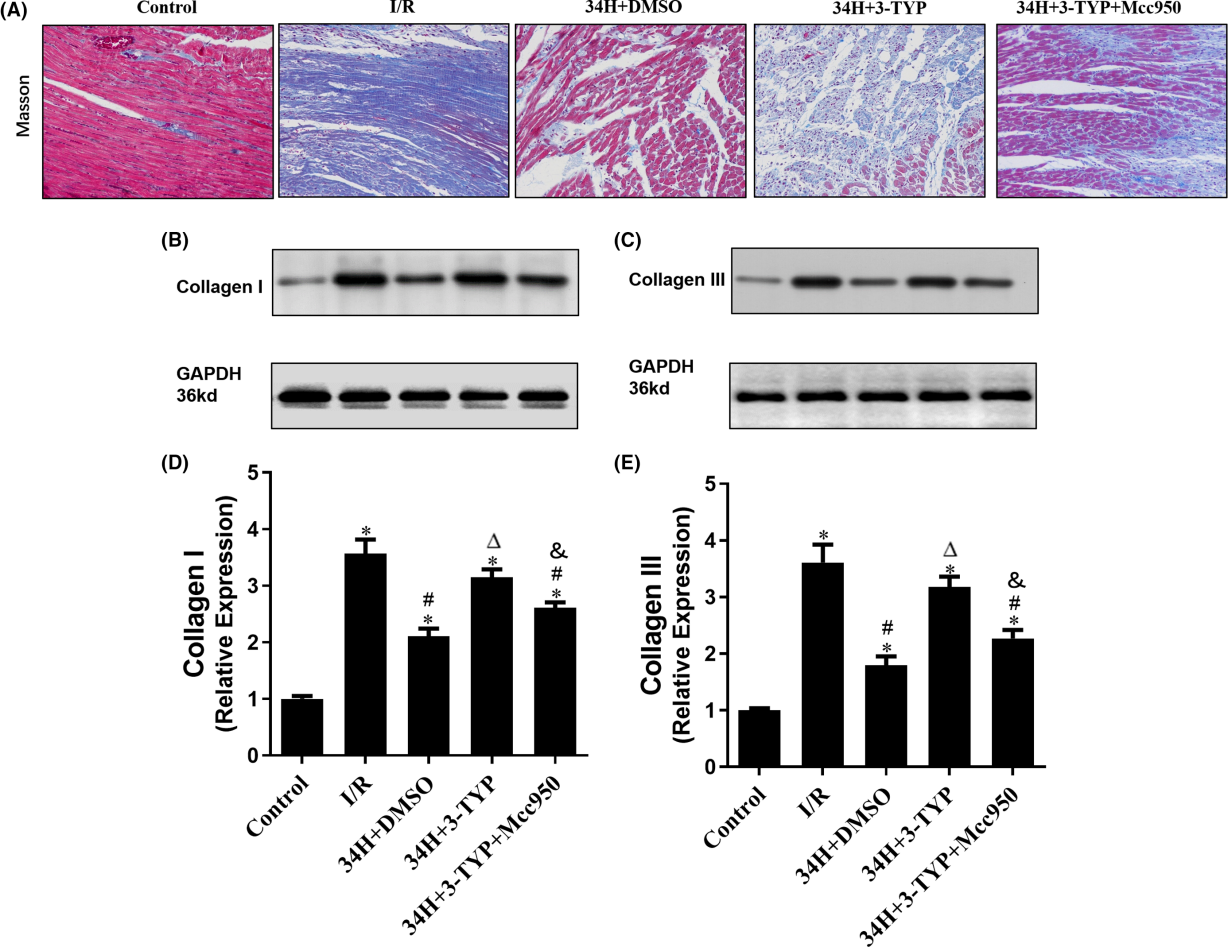
Masson staining and WB assay were applied to assess the extent of fibrosis in the heart tissue sections. Masson staining of cardiac fibrosis (A) and WB assay of cardiac fibrosis biomarkers, including collagen I (B, D) and collagen III (C, E), of the isolated heart in all groups. *p < 0.05 versus control group; #*p* < 0.05 versus I/R group; ^Δ^ *p* < 0.05 versus 34H+DMSO group. ^&^ *p* < 0.05 versus 34H+3‐TYP group.

Masson's trichrome staining was performed for histopathological analysis of the isolated heart models in all groups. Masson staining revealed severe fibrosis in the I/R group, with remarkably increased blue scar tissue at the edge of the infarct area compared to the Control group. The myocardial infarct symptoms and fibrosis response were ameliorated by TH therapy in the 34H+DMSO group, which was abolished by 3‐TYP in the 34H+3‐TYP group. It is worth noting that Mcc950 in the 34H+3‐TYP+Mcc950 group improved fibrosis, reversing the effect of 3‐TYP (Figure 3A).

Myocardial fibrosis was further validated via the WB assay, which was used to evaluate the expression of collagen I (Figure 3B,D) and collagen III (Figure 3C,E) in the isolated heart tissue of rat models in all groups. As presented in Figure 3B,C, the relative expression levels in each lane were normalized to the Control group in the first lane. The WB results from between‐group differences showed that the expression levels of collagen I and collagen III in the I/R group were significantly higher than those in the Control group (*p* < 0.05), and the 34H+DMSO group showed decreased collagen I and collagen III expression after I/R (*p* < 0.05). In contrast, rats treated with the SIRT3 inhibitor 3‐TYP in the 34H+3‐TYP group demonstrated considerably worsened fibrosis and dramatically increased protein levels of collagen I and collagen III compared to those in the 34H+DMSO group (*p* < 0.05), which could be alleviated by Mcc950 treatment in the 34H+3‐TYP+Mcc950 group compare with 34H+3‐TYP group (*p* < 0.05). These data suggest that TH downregulates the protein levels of collagen I and III in the heart post‐I/R to enhance the progression of fibrosis.

### 3.3 Therapeutic hypothermia attenuated myocardial apoptosis by enhancing SIRT3 expression of rat models after I/R injury

**FIGURE 4:**
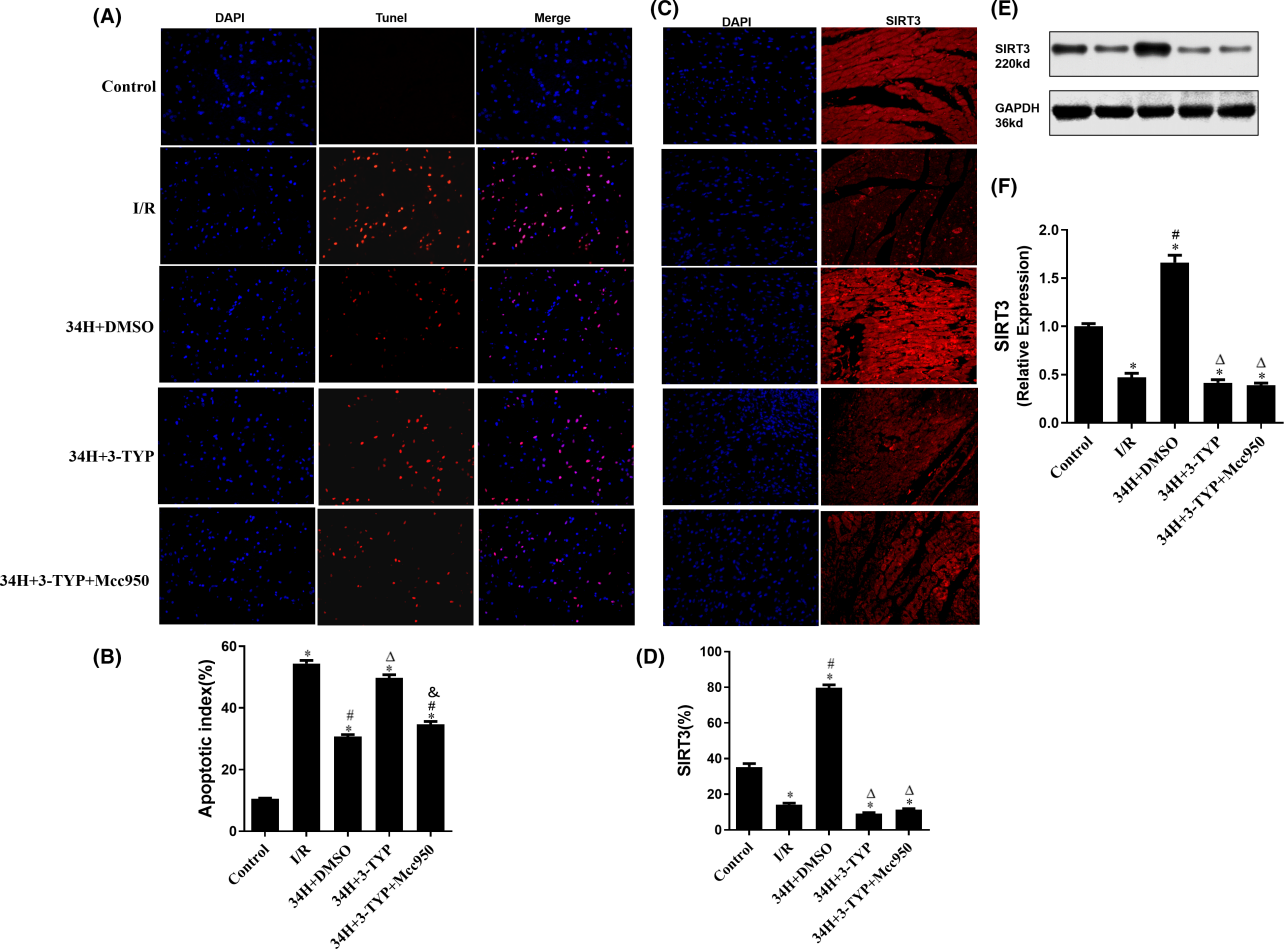
DAPI and TUNEL double staining was performed to identify apoptotic cells, and SIRT3 staining and WB assay were conducted to detect SIRT3 expression in the heart sections. (A, B) show the number of DAPI‐ and TUNEL‐positive cells and apoptotic index in all groups. (C, D) Representative images of SIRT3‐positive cells in heart sections in all groups. (E, F) Estimation of SIRT3 levels in each group was carried out using the WB assay. *p < 0.05 versus control group; #*p* < 0.05 versus I/R group; ^Δ^ *p* < 0.05 versus 34H+DMSO group. ^&^ *p* < 0.05 versus 34H+3‐TYP group.

Myocardial apoptosis was assessed by DAPI (blue) and TUNEL (red) double staining and quantified in isolated rat heart models after different treatments in each group (Figure 4A). Myocardial apoptosis was reduced in the Control group (cells labelled with red fluorescence were apoptotic cells), whereas an observably higher degree of red staining was observed in the I/R group (I/R), reflecting the larger number of apoptotic cells. A significantly lower apoptotic index was detected in the therapeutic hypothermia therapy group (34H+DMSO) than that in the I/R group (*p* < 0.05, Figure 4B). Furthermore, compared to the 34H+DMSO group, there was a significantly increased TUNEL‐positive cell count after adding 3‐TYP, an inhibitor of SIRT3, to the 34H+3‐TYP group (*p* < 0.05), which demonstrated a significantly higher apoptotic index than the 34H+3‐TYP+Mcc950 group (*p* < 0.05).

To further investigate the regulatory mechanism of TH intervention on myocardial cell apoptosis in rats after I/R, we detected the expression level of SIRT3 in the myocardial tissue of each group using SIRT3 staining (Figure 4C,D) and WB assay (Figure 4E,F). As shown in histograms 4D and 4F, the SIRT3 expression level in the myocardial tissue in the I/R group was significantly lower than that in the Control group (*p* < 0.05), whereas the SIRT3 level in the therapeutic hypothermia group was significantly higher than that in the Control group (*p* < 0.05) and the I/R group (*p* < 0.05), which could be abolished by the SIRT3 inhibitor 3‐TYP (*p* < 0.05). However, there was no statistical difference of the expression of SIRT3 compare the 34H+3‐TYP and 34H+3‐TYP+Mcc950 group. These findings indicate that the production of SIRT3 could increase dramatically after TH intervention pointing out that therapeutic hypothermia attenuates myocardial apoptosis post‐I/R, probably via enhancing the expression of SIRT3. Furthermore, the effect of 3‐TYP was inhibited by treatment with the NLRP3 inhibitor Mcc950 in the 34H+3‐TYP+Mcc950 group.

### 3.4 Therapeutic hypothermia inhibited myocardial inflammation induced by I/R via mediating the SIRT3/NLRP3 signalling pathway in the rat in vitro heart model

**FIGURE 5:**
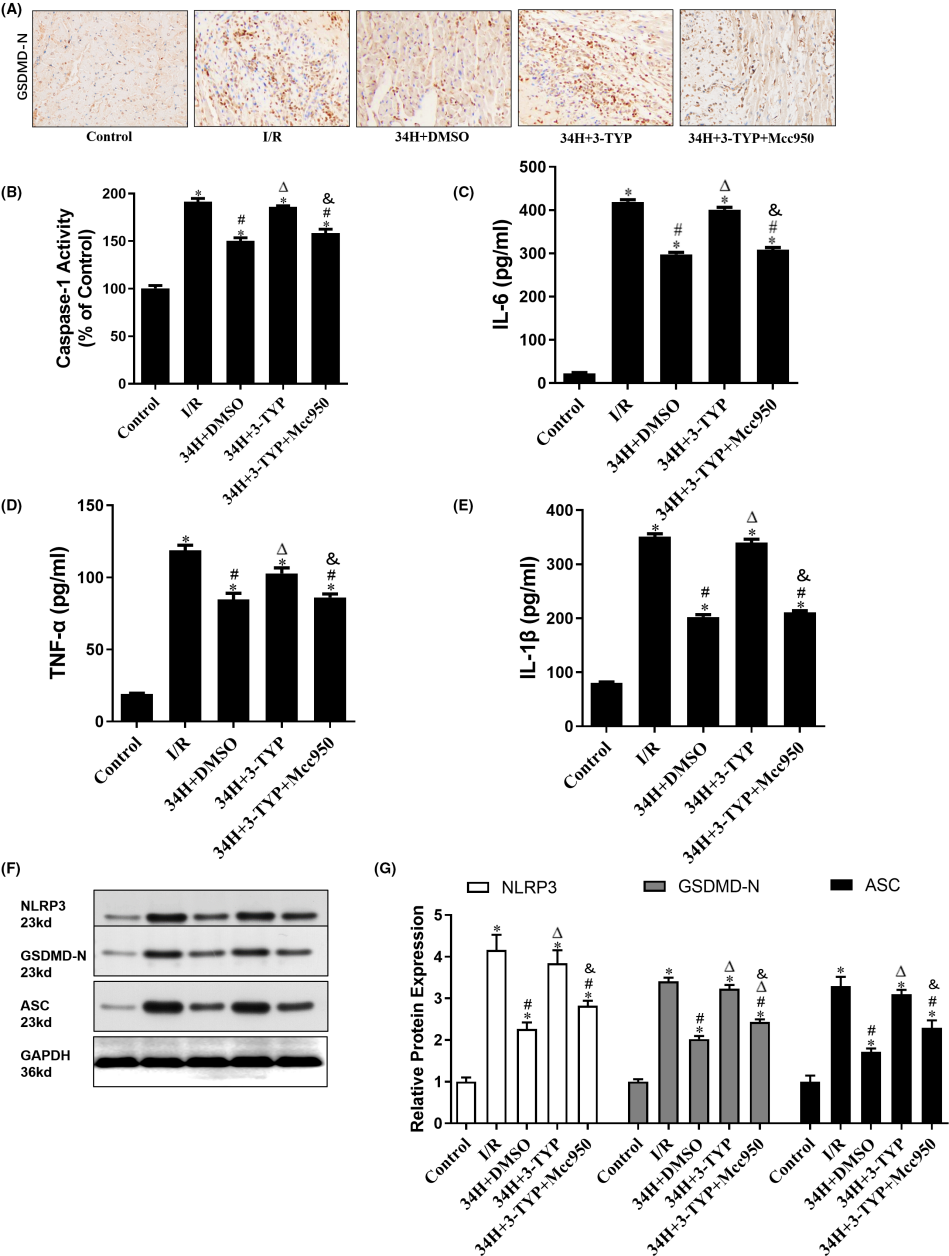
Therapeutic hypothermia alleviates inflammation caused by I/R. (A) Representative GSDMD‐N immunohistochemistry of all groups. (B–E) Immunohistochemistry for caspase‐1, Il‐6, TNF‐α and IL‐1β in all groups. (F, G) WB analysis of NLRP3, GSDMD‐N and ASC in all groups. *p < 0.05 versus control group; #*p* < 0.05 versus I/R group; ^Δ^ *p* < 0.05 versus 34H+DMSO group. ^&^ *p* < 0.05 versus 34H+3‐TYP group.

The anti‐inflammatory effects of TH were reflected by changes in the level of inflammatory cell infiltration in different groups using IHC (Figure 5A). On IHC, some myocardial cells in the Control group were stained yellow, indicating the expression of the inflammatory marker GSDMD‐N. Extensive yellow staining of myocardial cell cytoplasm was observed in the I/R group, indicating that GSDMD‐N production was particularly evident compared with the Control group (*p* < 0.05). However, after TH treatment, the yellow staining of the cytoplasm decreased markedly, indicating that the expression of GSDMD‐N in the 34H+DMSO group was significantly lower than that in the I/R group (*p* < 0.05). In addition, IHC analysis revealed that GSDMD‐N expression in the 34H+3‐TYP group was significantly higher than that in the 34H+DMSO group after treatment with the SIRT3 inhibitor 3‐TYP (*p* < 0.05) but reduced after treatment with the NLRP3 inhibitor Mcc950 in the 34H+3‐TYP+Mcc950 group (*p* < 0.05). Moreover, the expression levels of the inflammatory factors caspase‐1, IL‐6, TNF‐α and IL‐1β were determined (Figure 5B–E). In the I/R group, the expression of leak fluid caspase‐1, IL‐6, TNF‐α and IL‐1β was significantly increased compared to that in the Control group, which was significantly reversed in the 34H+DMSO group (*p* < 0.05). Consistent with previous changes in GSDMD‐N, the production of leak fluid caspase‐1, IL‐6, TNF‐α and IL‐1β in the 34H+3‐TYP group was higher than that in the 34H+DMSO group (*p* < 0.05) but lower than that in the 34H+3‐TYP+Mcc950 group (*p* < 0.05).

To further explore the mechanism of therapeutic hypothermia in inhibiting the inflammatory level of myocardial cells, we investigated the expression of components of the NLRP3 inflammasome. As shown in Figure 5F,G, the expression levels of NLRP3, GSDMD‐N and ASC in the I/R group were significantly higher than those in the Control group (*p* < 0.05) and 34H+DMSO group (*p* < 0.05), as revealed by Western blotting. In addition, the expression level of NLRP3, GSDMD‐N and ASC in the 34H+3‐TYP group was higher than that in the 34H+DMSO group (*p* < 0.05) and that in the 34H+3‐TYP+Mcc950 group (*p* < 0.05).

## 4 DISCUSSION

**FIGURE 6:**
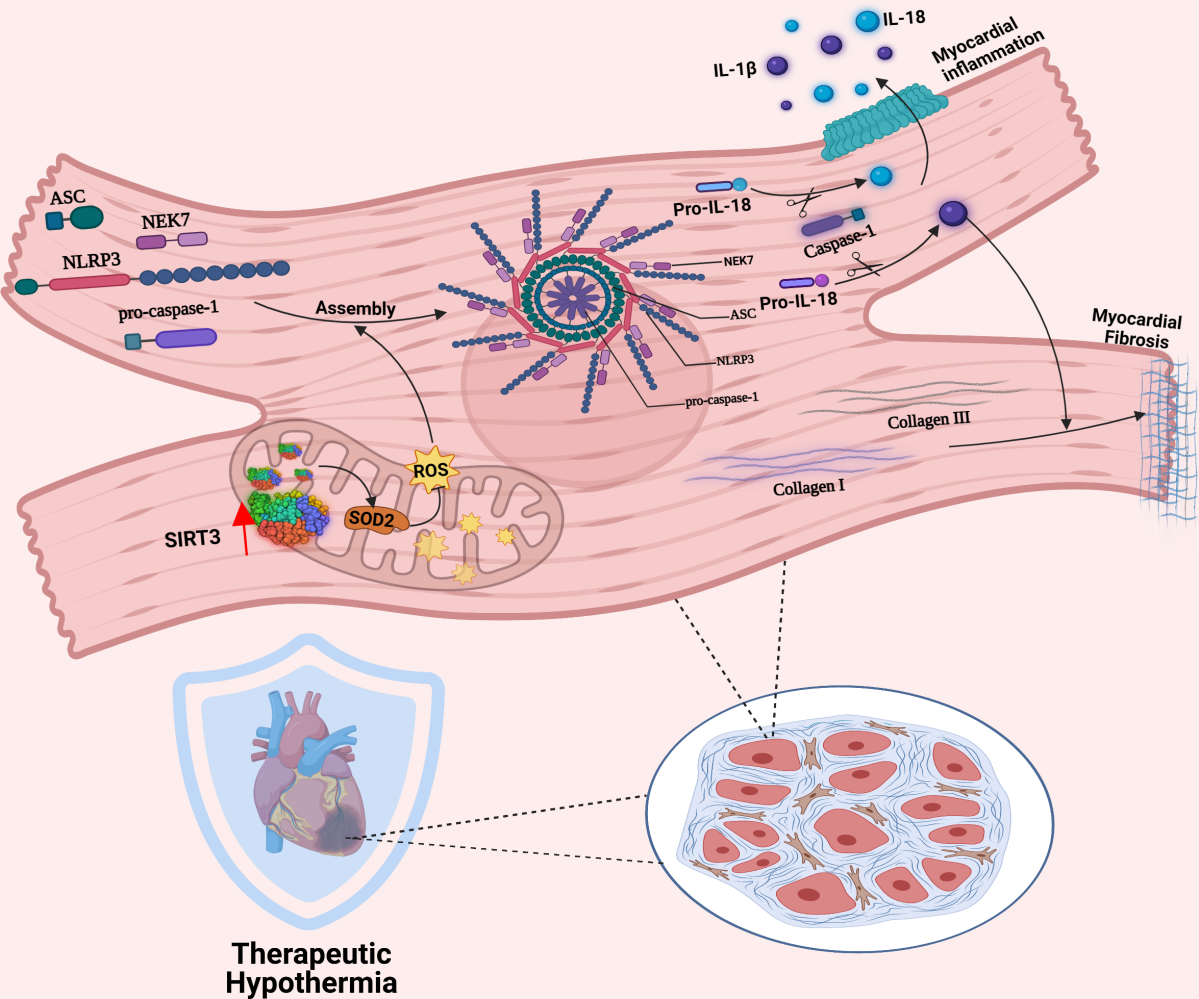
Mechanism of the SIRT3/NLRP3 signalling pathway modulating myocardial inflammation and fibrosis in cardiomyocytes post‐I/R of TH

In the present study, TH intervention at 34°C immediately after the start of reperfusion decreased myocardial infarct size, preserved cardiac function, attenuated myocardial apoptosis, and alleviated myocardial fibrosis and inflammation; thus, TH had significant cardioprotective effects. In addition, the cardioprotective effects of TH were reversed by the SIRT3 inhibitor 3‐TYP, whose reversal effects were abolished by the NLRP3 inhibitor Mcc950. These phenomena suggest that SIRT3 overexpression induced by TH may exert myocardial protection as an upstream regulatory signal for the NLRP3 inflammasome. Therefore, the data above suggest that the cardioprotective effects of TH against MIRI are partly mediated by the SIRT3/NLRP3 signalling pathway (Figure 6).

AMI damages the myocardium via two processes: ischaemia and subsequent MIRI.^17^ MIRI is an important pathophysiological phenomenon that occurs during MI. When cardiomyocytes are subjected to IRI, the lack of oxygen in cells leads to mitochondrial dysfunction and abnormal energy metabolism, leading to apoptosis and necrosis of tissue cells.^3^, ^18^ Previous evidence has shown that myocardial cell apoptosis, activation of inflammation and myocardial fibrosis are the pathological mechanisms related to MIRI induced by AMI.^4^, ^19^ In recent years, myocardial cell apoptosis, inflammation and myocardial fibrosis have been reported to alleviate MIRI.

Over the past few decades, various therapeutic strategies to ameliorate MIRI have been investigated, and some have been widely applied in clinical practice.^2^, ^20^, ^21^ Among these strategies, TH has attracted increasing attention in recent years because of its function and significance. Since its initial clinical use by Bigelow et al.^22^ in 1950, when the neuroprotective effects were confirmed during cardiac surgery, TH was spotlighted by the scientific community for its therapeutic potential in improving various diseases, including acute stroke,^23^ cardiac arrest,^24^ hypoxic–ischaemic coma,^25^ traumatic brain injury^26^ and neurological injury^27^; however, its clinical advantages for MIRI are still under investigation. Recently, accumulating research has been conducted on the protection of TH on the heart, especially in AMI; the earlier the temperature in the reperfusion area reaches the mild temperature range, the less the MI area will be.^28^, ^29^

SIRT3, a mitochondrial deacetylase, exerts therapeutic effects by inhibiting cardiac inflammation and fibrosis.^30^, ^31^ In the heart, SIRT3 is highly expressed and localized in the mitochondria, where it is essential for the regulation of metabolism and oxidative stress. Studies have demonstrated that SIRT3 overexpression in cardiomyocytes provides cardioprotective responses and highlights its anti‐fibrotic and anti‐inflammation effects.^30^, ^32^, ^33^

The NLRP3 inflammasome plays an essential role in the pathophysiological process of MIRI after NLRP3 receives exogenous danger and environmental stress signals.^34^ NLRP3 oligomerizes to form a wheel‐shaped signalling hub, which can exacerbate inflammatory reactions.^35^ The assembly and activation of the NLRP3 inflammasome trigger the activation of caspase‐1, thereby promoting the release of inflammatory cytokines IL‐1β and IL‐18 cleaved by caspase‐1 and can also lead to pyroptosis mediated by gasdermin D protein, worsening MIRI.^36^, ^37^ Notably, there is growing evidence that enhancing SIRT3 activity can inhibit the activation of the NLRP3 inflammasome, thereby ameliorating the inflammatory insult.^38^, ^39^, ^40^

Building on previous data, we first explored the hypothesis that TH therapy attenuates MIRI by activating SIRT3 expression and subsequently inhibiting the NLRP3 inflammasome cascade. We treated isolated rat heart models with TH and attempted to elucidate the pathophysiological mechanism of TH intervention in MIRI. TTC staining revealed that TH markedly ameliorated the cardiac infarction. In HE staining, pathological changes in myocardial fibres, such as disorderly arrangement and marked breakage, were observed in the I/R group, which could be reversed by TH intervention. On DAPI and TUNEL double staining, significantly reduced myocardial TUNEL‐positive cell count following TH intervention was observed. Moreover, in WB analysis, the expression levels of inflammatory factors IL‐6, TNF‐α and IL‐1β, cleaved caspase‐1 protein, and collagen I and collagen III in isolated heart models post‐I/R after TH intervention were prominently lower than those in myocardial cells in the I/R model, indicating that TH intervention exerted anti‐apoptotic, anti‐inflammatory and anti‐fibrotic effects on myocardial cells after I/R injury. To further explore the underlying molecular mechanism of myocardial protection by TH therapy, we conducted a state‐of‐the‐art review and literature search on the mechanisms of inflammation and fibrosis and found that the SIRT3/NLRP3 signalling pathway plays a central role in mediating MIRI. 3‐TYP is a SIRT3‐selective inhibitor. Mcc950 can selectively block NLRP3 inflammasome activation. Therefore, based on this groundbreaking breakthrough point, we brought new insights into the hypothesis that TH therapy may inhibit inflammation and fibrosis in myocardial cells by blocking the SIRT3/NLRP3 signalling pathway. Determination of the expression levels of SIRT3 protein and NLRP3 inflammasome components in isolated heart models revealed that SIRT3 levels in myocardial sections were dramatically increased in the 34H+DMSO group, whereas those of NLRP3 inflammasome components were significantly decreased after TH intervention. Our breakthrough of TH targeting SIRT3 in cardiomyocytes opens a novel avenue of therapeutic approach regarding utilizing TH therapy to activate the SIRT3/NLRP3 signalling pathway to cure patients post I/R and is an important complement to the role of the SIRT3/NLRP3 signalling pathway in myocardial inflammation and fibrosis.

Although a breakthrough discovery for TH could ameliorate MIRI by mediating the SIRT3/NLRP3 signalling pathway, the exact mechanism underlying SIRT3 overexpression induced by TH regulating NLRP3 in MIRI is unknown. Further research is required to test the biological role and molecular mechanism of SIRT3 in NLRP3 inflammasome progression. In addition, we acknowledge that our work has some limitations. First, we conducted this study to investigate the effects of TH in vitro using the Langendorff isolated cardiac perfusion model, which cannot fully replicate physiological conditions in vivo exhibiting some limitations in assessing I/R damage. Therefore, further studies are needed to confirm whether TH intervention may also prove useful in vivo. Second, we utilized well‐established and specific inhibitors 3‐TYP and Mcc950, but no inhibitors were entirely specific; SIRT3‐knockout and/or NLRP3‐knockout mice may provide more convincing evidence.

With the development of TH at 34°C for MIRI, it may be possible to take advantage of this unique tool that significantly decreases the expression of SIRT3 and increases that of NLRP3 inflammasome in MIRI to develop the drugs that mimic the agonists of SIRT3 in combination with TH at 34°C for clinical therapy in patients with AMI. However, further research is necessary to investigate the long‐term effects of TH on MIRI. Further clinical studies are needed to validate this conclusion.

## 5 CONCLUSION

In summary, we explored the cardioprotective effects and potential mechanisms of TH at 34°C in a rat isolated heart model against I/R injury and demonstrated that myocardial ischaemia–reperfusion exacerbated myocardial inflammation and fibrosis, aggravated myocardial apoptosis, and worsened cardiac function, which all could be ameliorated by TH. Furthermore, we noted that TH intervention exerted its cardioprotective effect possibly by mediating the SIRT3/NLRP3 signalling pathway, which has important clinical ramifications for applying TH therapy to the treatment of patients with I/R.

## AUTHOR CONTRIBUTIONS

**Jing Zhang:** Conceptualization (equal); investigation (equal); methodology (equal); project administration (equal). **Yimei Lu:** Conceptualization (equal); investigation (equal); methodology (equal); project administration (equal). **Peng Yu:** Data curation (equal); methodology (equal); resources (equal); validation (equal). **Zhangwang Li:** Data curation (equal). **Yang Liu:** Writing – original draft (equal). **Jun Zhang:** Writing – original draft (equal). **Xiaoyi Tang:** Writing – original draft (equal).

## FUNDING INFORMATION

This work was supported by the National Natural Science Foundation of China [Nos. 81760338 and 81360285 to S.Y.].

## CONFLICT OF INTERESTS

The authors declare no conflict of interest.

## Data Availability

The data that support the findings of this study are available from the corresponding author's resealable request.
